# Respiratory patterns in spontaneously breathing near-term lambs delivered by caesarean section under spinal anaesthesia

**DOI:** 10.3389/fped.2023.1273136

**Published:** 2023-10-09

**Authors:** I. M. Davies, K. J. Crossley, E. V. McGillick, I. Nitsos, K. Rodgers, A. Thiel, V. A. Zahra, A. B. te Pas, S. B. Hooper

**Affiliations:** ^1^ The Ritchie Centre, Hudson Institute of Medical Research, Melbourne, VIC, Australia; ^2^ The Department of Obstetrics and Gynaecology, Monash University, Melbourne, VIC, Australia; ^3^ Division of Neonatology, Department of Pediatrics, Leiden University Medical Center, Leiden, Netherlands

**Keywords:** newborn, breathing patterns, respiratory distress, caesarean section, newborn lambs

## Abstract

**Introduction:**

The transition to newborn life has typically been studied in intubated and mechanically ventilated newborn lambs delivered via caesarean section (CS) under general anaesthesia. As a result, little is known of the spontaneous breathing patterns in lambs at birth, particularly those at risk of developing respiratory distress (RD). We have developed a method for delivering spontaneously breathing near-term lambs to characterise their breathing patterns in the immediate newborn period.

**Methods:**

At 137–8 days gestation (2–3 days prior to delivery; term ∼147 days), fetal lambs (*n* = 7) were partially exteriorised for instrumentation (insertion of catheters and flow probes) before they were returned to the uterus. At 140 days, lambs were delivered via CS under light maternal sedation and spinal anaesthesia. Lambs were physically stimulated and when continuous breathing was established, the umbilical cord was clamped. Breathing patterns were assessed by measuring intrapleural and upper-tracheal pressures during the first four hours after birth.

**Results:**

Newborn lambs display significant heterogeneity in respiratory patterns in the immediate newborn period that change with time after birth. Seven distinct breathing patterns were identified including: (i) quiet (tidal) breathing, (ii) breathing during active periods, (iii) breathing during oral feeding, (iv) tachypnoea, (v) expiratory braking manoeuvres, (vi) expiratory pauses or holding, and (vii) step changes in ventilation.

**Conclusions:**

We have described normal respiratory behaviour in newborn lambs, in order to identify respiratory behaviours that are indicative of RD in term newborn infants.

## 1. Introduction

Fetal sheep have been used to study the unique physiology of the fetus, with the most critical insights provided by studies in unanaesthetised chronically catheterised fetal sheep (1). However, ethical and logistical issues associated with studying lambs during and immediately after birth has restricted studies examining the physiological transition at birth to lambs delivered by caesarean section (CS) under maternal general anaesthesia. As a result, these lambs are born anaesthetised and so must be intubated and mechanically ventilated to survive. These studies have provided critical information on the cardiorespiratory changes at birth that are unaffected by anaesthesia, which include the mechanics of lung aeration (2) and the physiological effects of cord clamping before and after lung aeration (3). However, they have provided little to no information on the factors regulating respiratory control in spontaneously breathing newborns at the time of birth.

Non-invasive respiratory support, usually administered via a facemask, has become the mainstay of newborn resuscitation because the alternative, intubation and mechanical ventilation, is more invasive and potentially injurious to the lung. However, the recent discovery that newborns close their glottis if they become apneic after birth (4) has highlighted the fact that innate respiratory control in newborns immediately after birth more closely resembles that of a fetus than an infant/adult. While glottic closure can prevent or seriously impede the administration of non-invasive ventilation, the simple solution is to stimulate the baby to breathe so that the glottis will open. As such, there is a pressing need for greater understanding of the factors that promote newborn breathing, whilst avoiding factors that inhibit breathing (e.g., transient hypoxia).

Much research has focussed on the respiratory management of preterm infants at birth, but relatively few studies have focused on near-term and term infants who subsequently develop respiratory distress (RD) in the first few hours after birth. In contrast to preterm infants, these infants have structurally and functionally mature lungs, but develop RD that is characterised by tachypnea, grunting, expiratory braking, and chest wall retraction (5). However, as many of these behaviours are also thought to characterise respiratory patterns that normally occur at birth (6), when normal becomes abnormal is entirely subjective, making it difficult to identify infants developing RD.

In this study, we aimed to deliver spontaneously breathing near-term newborn lambs via CS under spinal anaesthesia and characterise the respiratory patterns of near-term newborns for the first four hours after birth.

## 2. Materials and methods

### 2.1 . Ethical approval

All experimental procedures were approved by the Monash Medical Centre Animal Ethics Committee and Monash University. All experiments were conducted in accordance with the National Health and Medical Research Council (NHMRC) of Australia code of practice for the care and use of animals for scientific purposes (7). Methodological reporting is provided as per the relevant ARRIVE guidelines (8).

### 2.2. Fetal instrumentation prior to CS delivery

At 126 days gestation (term ∼147 days), ewes were administered medroxyprogesterone acetate (150 mg; i.m. injection) to prevent spontaneous labour. Sterile surgery was performed on pregnant Merino X Border-Leicester ewes at 137–8 days gestation, 2–3 days prior to CS delivery. General anaesthesia was induced (*n* = 7) using sodium thiopentone (Pentothal; 1 g in 20 ml; i.v. bolus) and maintained with inhaled 1.5%–2.5% isoflurane (Isoflow, Abbot) in room air/oxygen as previously described (9). The fetal head and neck were exposed via hysterotomy to implant vascular catheters into the left common carotid artery (blood sampling) and the left internal jugular vein (post-surgical antibiotic administration and drug administration following delivery). A blood flow probe (4 mm ultrasonic; Transonic Systems, Ithaca, New York, USA) was placed around the left pulmonary artery via a left thoracotomy and a saline-filled latex balloon tipped catheter was also placed within the intrapleural space (10). A small polyvinyl catheter was implanted into the upper trachea, by insertion between two rings, leaving the tip approximately 3 cm below the larynx; as it was small in diameter (2.6 mm O.D.), relative to the trachea, it did not impede normal lung liquid or gas flow post-surgery. A blood flow probe (3 mm ultrasonic; Transonic Systems, Ithaca, New York, USA) was also placed around the right common carotid artery and large bore amniotic catheter was implanted to deliver antibiotics post-operatively. Following instrumentation, the fetus was returned to the uterus, which was closed and the catheters exteriorised via the ewe's flank. Antibiotics were administered on the day of surgery and once daily for 2–3 days post-operatively (Cefazolin, AFT Pharmaceuticals; maternal i.v. 1,000 mg; fetal i.v. 100 mg; intra-amniotic 400 mg). Ewes also received analgesia via transdermal fentanyl patch (75 *μ*g/hour) during the post-operative period.

### 2.3. Maternal sedation and spinal anaesthesia during CS delivery

At 140 days gestation (term ∼147 days), ewes were positioned upright in a purpose-built sling trolley and amniotic fluid volumes were passively drained to simulate membrane rupture (11). Recordings of fetal physiological parameters (pulmonary and carotid blood flow, tracheal pressure, and intrapleural pressure) commenced prior to (∼20 min) and continued throughout delivery, using a data acquisition system (Powerlab ADI, Sydney, Australia). Intrapleural and tracheal pressure was measured via pressure transducer (TNF-R, BD Dtxplus™, DT, Mumbai) and the pressure signal amplified via quad bridge amplifier (ADInstruments, Sydney, Australia). Fetal arterial blood samples were taken immediately prior to delivery for blood gas analysis. Lambs were delivered via CS under spinal anaesthesia as previously described (12). Ewes were briefly (3–10 min) sedated (Propofol 1%; 20–40 mg bolus; i.v.) to administer the spinal anaesthesia (Lignocaine 2%; 0.1 ml/kg) into the subarachnoid space of the lumbar-sacral intersection. This induces a complete spinal block of the lower body, which was verified by the absence of reflexes and muscle tone in the hind legs. To minimise respiratory depression caused by propofol crossing the placenta, CS delivery was delayed until after the ewe had fully recovered (∼10 min). The ewe was then mildly sedated (Midazolam; 1 mg/kg/hr; i.v. infusion) and given oxygen via a nasal cannula (5 L/min, 100% O_2_). The ewe's hindquarters were then moved into a lateral recumbent position and as much fetal lung liquid as possible was drained directly via the tracheal catheter (39.7 ± 11.7 ml). The lamb was then exteriorised through a lateral (right sided) incision within the ewe's abdomen, taking care to ensure that the umbilical cord remained intact.

### 2.4. Management during fetal to neonatal cardiorespiratory transition

**Figure 1:**
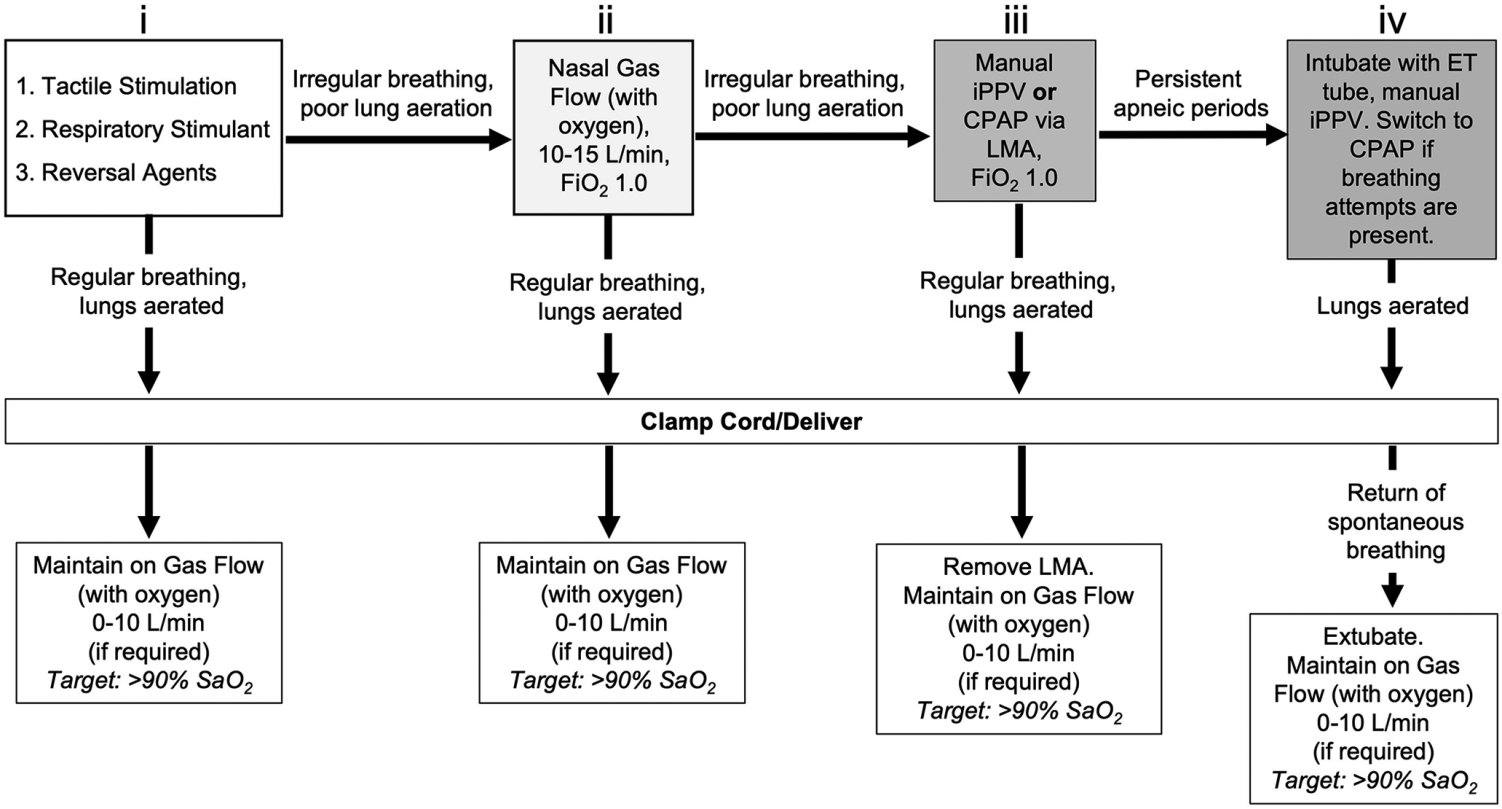
Resuscitation protocol during caesarean section delivery of lambs under spinal anaesthesia with maternal sedation. To stimulate breathing efforts, lambs initially received (i) tactile stimulation in conjunction with caffeine therapy (20 mg/kg, i.v. bolus) and reversal agents for maternally administered sedatives (0.01 mg/kg Flumazenil and 0.01 mg/kg Naloxone; i.v. bolus). If poor respiratory efforts were present, (ii) non-invasive support was provided by gas flow with oxygen supplementation via nasal cannula (10-15 L/min, 100% O_2_). If respiratory efforts did not improve, (iii) additional support was provided via manual intermittent positive pressure ventilation (iPPV) delivered by laryngeal mask airway (LMA, V-gel® Advanced, size R6; 100% O_2_) using an infant t-piece resuscitator (NeoPuff™, Fisher & Paykel, Auckland, New Zealand) with peak inflation pressures (PIP) of 35 cm H_2_O and positive end expiratory pressure (PEEP) of 5 cm H_2_O. Inadequate respiratory efforts warranted (iv) endotracheal (ET) intubation (MD03345-4.0, cuffed, Portex Ltd, England) and subsequent delivery of volume-guarantee iPPV in assist control mode using a back-up rate of 30 breaths per minute. Once respiratory efforts were present, respiratory support was switched back to continuous positive airway pressure (CPAP; 5-8 cm H_2_O, 100% O_2_). Once consistent breathing was achieved, the umbilical cord was clamped and the lamb delivered. Lambs delivered while intubated (LMA or ET tube) were extubated following return of spontaneous breathing. All lambs were maintained after cord clamp on gas flow with oxygen supplementation *via* nasal cannula (if required; 0-10 L/min) with an arterial oxygen saturation (SaO_2_) target of >90%.

**Figure 2:**
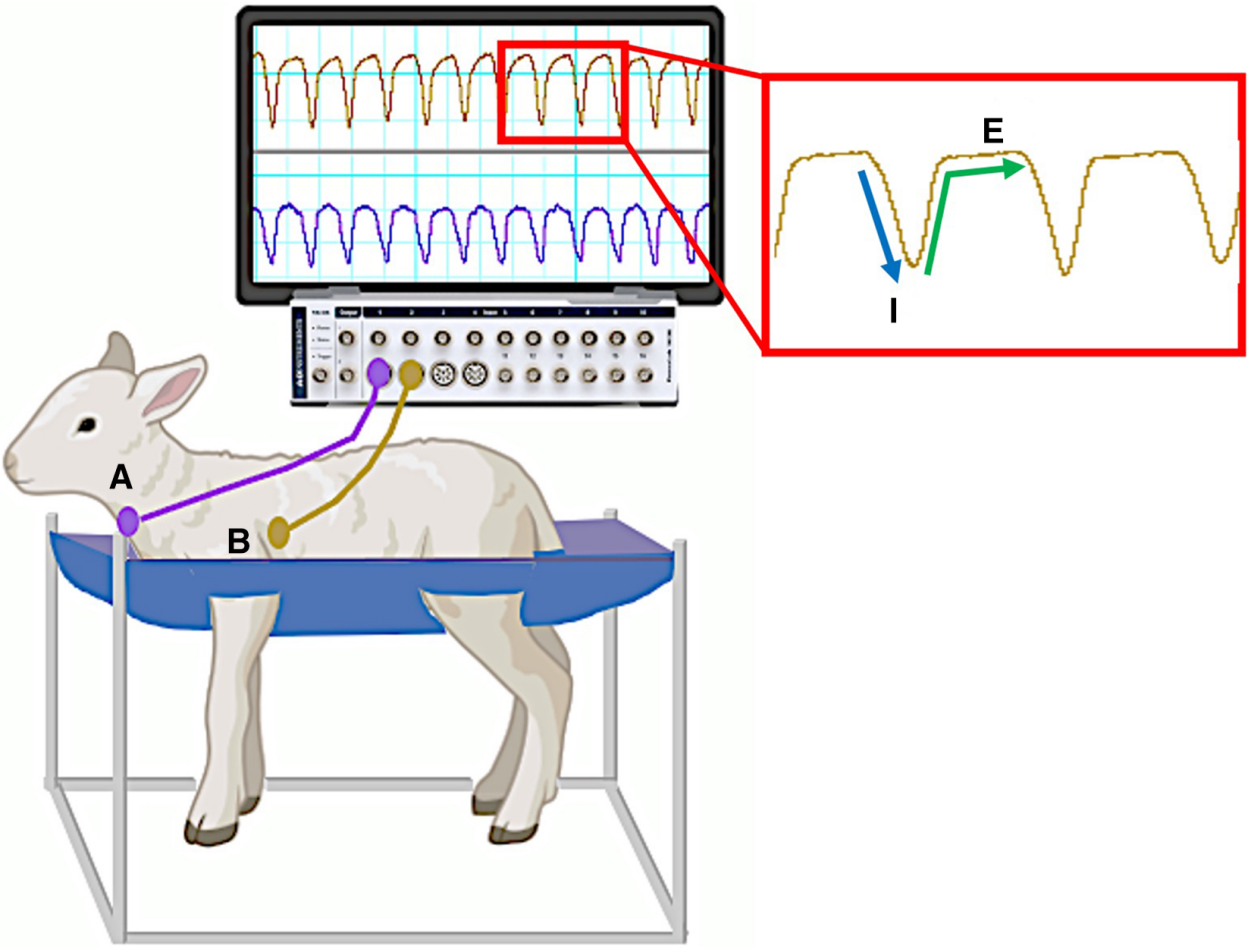
Custom sling designed to hold instrumented spontaneously breathing newborn lamb. Monitoring devices in instrumented lambs were connected to pressure transducers and recorded via a data acquisition system running LabChart8 software (Powerlab; ADInstruments, Sydney, Australia). Breathing movements were identified by measuring changes in intratracheal (**A**) and intrapleural (**B**) pressure (cmH_2_O) throughout each breathing cycle. A characteristic digital breathing trace shows a negative change in pressure during inspiration (**I**) followed by a return to baseline pressure during expiration (**E**) Additional instrumentation including carotid artery/jugular vein catheters and pulmonary/carotid artery flow probes are not shown. *Lamb graphic obtained from* Biorender.com.

Following delivery, the lamb was given oxygen via nasal prongs (5–10 L/min, 100% O_2_) and was physically stimulated to encourage spontaneous breathing whilst the umbilical cord remained intact. This is referred to as physiological based cord clamping and is dependent on pulmonary blood flow (PBF) increasing before the cord is clamped; we measured PBF to provide real-time feedback on the state of transition (3, 13). As midazolam also crosses the placenta, lambs were also given Flumazenil (0.01 mg/kg; i.v.) to block any depressive effects of maternally administered midazolam on respiratory drive. Similarly, Naloxone (0.01 mg/kg; i.v.) was given to the lamb to counter the effects of any remaining fentanyl following surgery. In addition to tactile stimulation, caffeine (20 mg/kg; i.v. bolus) was given to stimulate breathing, which was followed by a second dose if required. Lambs received a gradual escalation in care (no set time between escalations) and escalations were based on physiological outcomes such as regular breathing efforts, oxygenation, heart rate and increases in pulmonary blood flow (see Figure 1 for full resuscitation protocol). This commenced with increasing oxygen flow via nasal cannulae (flow rate increased to 10–15 L/min) and then escalated to; insertion of a laryngeal mask (LMA; v-gel® advanced size R6, Docsinnovent Ltd., UK) and application of continuous positive airway pressure (CPAP); positive pressure ventilation (Neopuff) via the LMA using a peak inflation pressure (PIP) of 35 cmH_2_O and a positive end expiratory pressure (PEEP) of 5 cmH_2_O; and finally to intubation (MD03345–4.0, cuffed, Portex Ltd, England) and positive pressure mechanical ventilation (Draeger Babylog 8,000 + in assist control) if breathing effort was still insufficient and pulmonary blood flow had not increased. If intubated, lambs were extubated as soon as possible after they commenced breathing. Once consistent breathing was established, the umbilical cord was clamped and lambs were transferred to a custom-made sling under a radiant heater (Figure 2). Following delivery of the lamb, ewes were euthanised with an i.v. overdose of sodium pentobarbitone (Virbac Pty. Ltd., Peakhurst, Australia).

### 2.5. Optimising respiratory drive after cord clamping

Several techniques were used to establish and optimise respiratory drive in the immediate newborn period, which included (i) applying tactile stimulation to the lamb by rubbing/drying its limbs and trunk (ii) the administration of respiratory stimulants such as caffeine and (iii) nasal gas flow (up to 15 L/min) with oxygen supplementation. We also routinely suctioned the lamb's nasopharynx and oropharynx after birth to clear the upper respiratory tract of mucal secretions which, in our experience, can occur following prenatal surgery on the upper trachea in sheep.

### 2.6. Monitoring and support of spontaneously breathing lambs after delivery

Recordings of breathing movements (measured by pressure changes in the tracheal and intrapleural balloon tipped catheters), arterial blood flows (pulmonary and carotid arteries) and pressures as well as other physiological parameters continued for four hours after birth (Powerlab, ADInstruments, Castle Hill, Australia). Arterial blood gas samples (0.3 ml) were collected every 5 min for the first 30 min after umbilical cord clamping, every 10 min until one hour and then every 20 min for the remaining four hours. Alveolar-arterial differences in oxygen (AaDO_2_) values were calculated at the same timepoints from blood gas measures as previously described (14). Nasal gas flow with oxygen supplementation were provided (flow rate restricted to 0–10 L/min) as required to maintain arterial oxygen saturations >90%. Lamb core body temperatures were maintained at ∼39 °C and lambs received regular enteral feeds with formula (Profelac Shepard milk replacement; ProviCo Nutrition; Victoria, Australia) as well as a continuous infusion (glucose 5%, 6 ml/kg/hr, i.v.). Following the onset of regular stable breathing, environmental stimuli (bright lights, loud noises, physical touching of the lamb) were minimised to avoid exciting the lamb and disturbing the breathing recordings. At the conclusion of the experiment, all lambs were euthanised using an i.v. overdose of sodium pentobarbitone (Virbac Pty. Ltd., Peakhurst, Australia).

### 2.7. Breathing classification and analysis

Using the physiological recordings, every minute of breathing was examined (by IMD) and classified into one of seven breathing patterns:

(i) quiet (tidal) breathing was defined as stable breathing with a consistent rate (40–69 breaths/min), waveform shape, and amplitude.
(ii) breathing while active or moving (confirmed visually). This occurred during exercise (rapid leg movements/shaking), experimenters undertaking procedures (e.g. adjusting monitoring devices or other physical stimulation), or vocalisation/bleating and sneezing,
(iii) breathing during oral feeds (confirmed visually),
(iv) tachypnea, defined as stable breathing with a consistent rate (≥ = 70 breaths/min), waveform shape and amplitude.
(v) expiratory pauses or expiratory holds,
(vi) expiratory braking manoeuvres characterised as either (a) grunting, (b) late-expiratory braking that was followed by the completion of expiration before the immediate start of inspiration, or (c) diaphragmatic braking,
(vii) step changes in ventilation.

To be counted as one of these defined breathing patterns, the duration of the displayed pattern had to be >30 s. Examples of each breathing pattern are provided in Table 1.

**Table 1:** Examples of respiratory patterns in spontaneously breathing near-term newborn lambs delivered by caesarean section.

Breathing pattern	Definition	Example
i. **Quiet** (tidal) breathing	Consistent breathing rate, waveform shape, and amplitude with respiratory rates between 40–69 breaths/min.	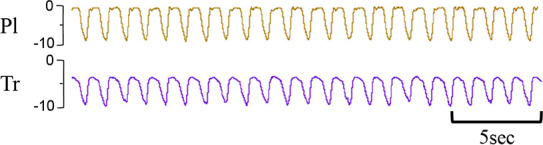
ii. Breathing while **Active**	Breathing during exercise (rapid leg movements/shaking), performing procedures (e.g. adjusting monitoring devices or other physical stimulation), or vocalisation/bleating and sneezing.	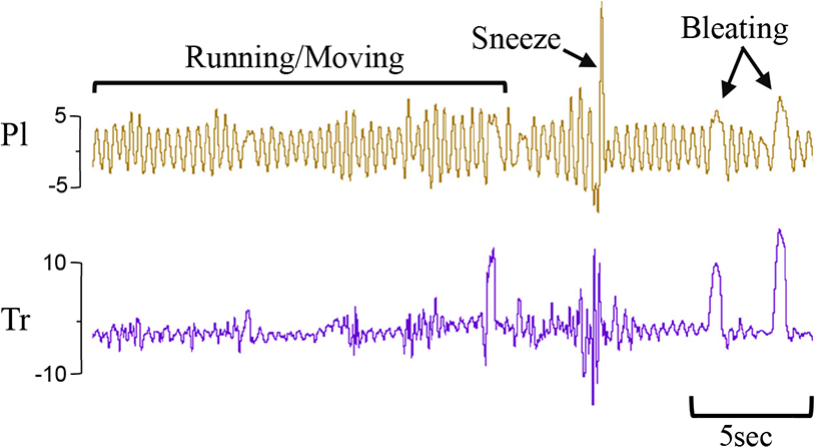
iii. Breathing during oral **Feeding**	Breathing patterns during oral feeding.	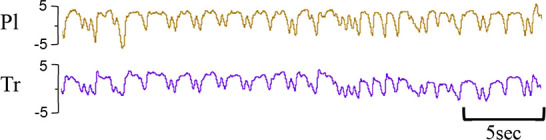
iv. **Tachypnea**	Consistent breathing rate, waveform shape, and amplitude with respiratory rates ≥ 70 breaths/min.	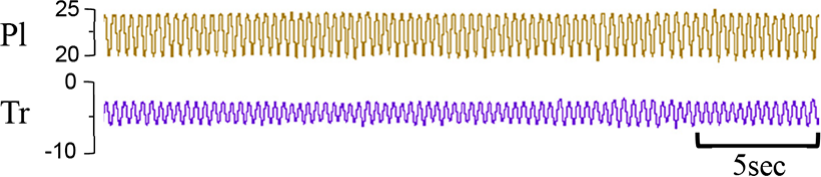
v. Expiratory **Pause** or Hold	Lengthening of the expiratory phase at end expiration (lung volume remains at FRC; black brackets).	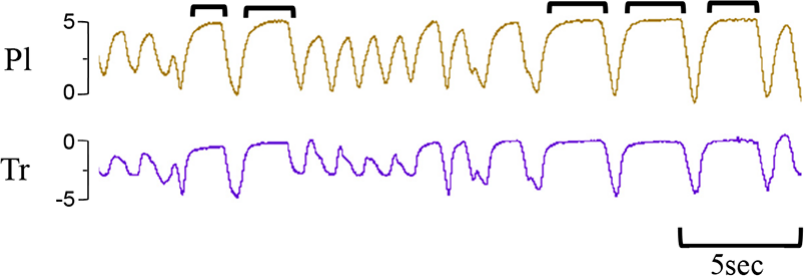
vi. Expiratory **Braking** Manoeuvres	Breathing maneuvers that extend the expiratory phase of a breath cycle. (a) Grunting: narrowing of the glottis during mid-expiration, denoted by an increase in pressure within the upper airway (black arrows; Tr) and lengthening of the expiratory phase. (b) Late-expiration braking: the cessation of flow during late expiration, followed by the completion of expiration immediately prior to the next inspiration (black brackets). (c) Diaphragmatic Braking: contraction of the diaphragm during expiration (black arrows).	(a) Grunting 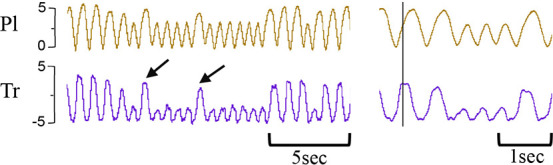 (b) Late-expiration braking 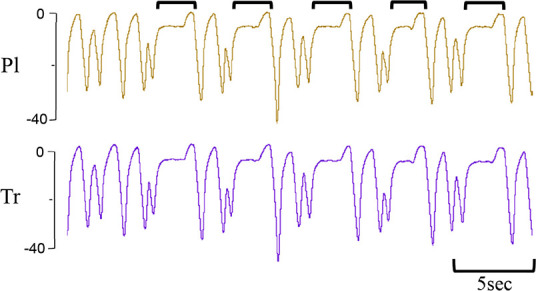 (c) Diaphragmatic braking 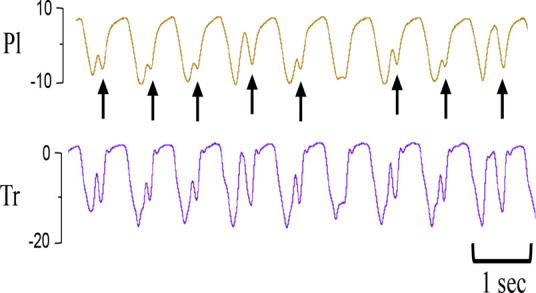
vii. **Step changes** in ventilation	Regular changes in respiratory rates and breath depth when lambs are relaxed or inactive. (Example displays four distinct changes in respiratory rate and breath depth, indicated by black dotted lines. These step changes commonly repeat in episodes that last between 5–20 minutes).	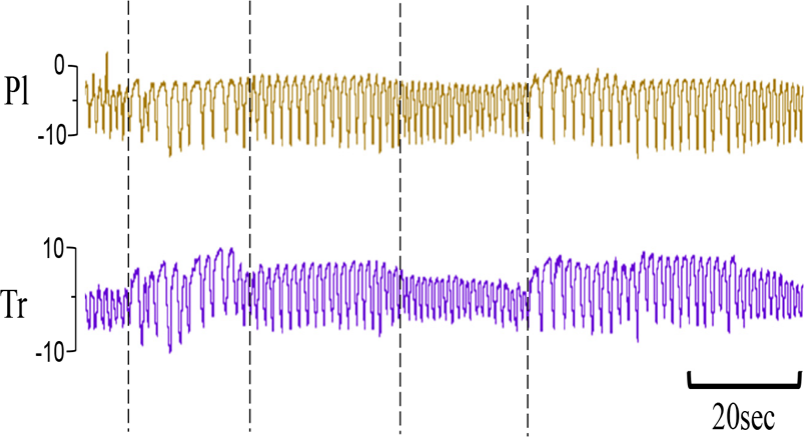

Breathing movements were measured by detecting changes in pressure (cmH_2_O) within the upper trachea (Tr) and intrapleural space (Pl) within the thorax throughout the breathing cycle. Inspiration is downward. FRC, functional residual capacity; Pl, intrapleural pressure waveform; Tr, intra-tracheal pressure waveform.

Additional breathing events including sighs and isolated post-inspiratory diaphragmatic contractions were also identified and classified into subtypes where appropriate.

### 2.8. Statistical analysis

All statistical analyses were performed with Prism v9 (GraphPad Software, San Diego, CA). All data were tested for normality (Shapiro-Wilk normality test). Continuous variables (blood gas values, fraction of inspired oxygen and alveolar-arterial difference in oxygen) that were normally distributed are presented as mean ± SEM and variables that were not normally distributed are presented as median (IQR). Breathing pattern proportions and events are presented as mean ± SEM. To understand the incidence and distribution of spontaneous breathing patterns and isolated breathing events during the first four hours after delivery, we investigated (i) the total proportion of time spent performing each breathing pattern or total number of isolated events over the duration of the experiment and (ii) the proportion of time spent performing each breathing pattern or total number of isolated events from cord clamping to 30 min, 30–60 min, and each hour thereafter and (iii) changes in the frequency of each breathing pattern or total number of isolated events across the experiment. Breathing patterns and data for isolated breathing events were analysed using one-way repeated measures ANOVA followed by Holm-Sidak *post hoc* test for multiple comparisons. Subtype of sighs were compared using Student's paired t-test (Type 1 vs. Type 2). *p *≤ 0.05 was considered statistically significant.

## 3. Results

### 3.1. Baseline characteristics and respiratory support prior to delivery

All lambs were delivered at the same gestational age and had normal fetal blood gas status prior to delivery (Table 2). Achieving consistent stable breathing on the umbilical cord took on average 6.6 ± 1.7 min, as measured from the time of first breath to the time of cord clamping. The most common form of respiratory support required to achieve stable breathing was nasal oxygen (Table 2; *n* = 5). One animal required endotracheal intubation and a short period of mechanical ventilation during the first minutes after exteriorisation (before cord clamping) due to inconsistent breathing and periods of apnea. One animal did not require any respiratory support prior to cord clamping (Table 2).

**Table 2:** Baseline characteristics, fetal blood gas values and respiratory support provided to near-term newborn lambs during resuscitation on the umbilical cord.

Measure	Results
Number of lambs	7
Male: female	4:3
Body weight (kg)	4.90 ± 0.16
Gestational age at delivery (d)	140 ± 0
Fetal blood gas values:	
pH	7.40 ± 0.00
pCO_2_ (mmHg)	49.5 ± 1.71
pO_2_ (mmHg)	18.6 ± 1.19
SaO_2_ (%)	53.4 ± 3.71
Lactate (mmol/L)	1.80 ± 0.30
Acid-base excess (mmol/L)	6.02 ± 1.04
Time from first breath to cord clamp (mins)	6.64 ± 1.67
Caffeine administration (dose = 20 mg/kg)	
0 doses	2/7
1 dose	2/7
2 doses	3/7
Maximum respiratory support required during resuscitation (stepwise escalation):	
None	1/7
Nasal oxygen	5/7
Laryngeal mask + iPPV on umbilical cord	0/7
Endotracheal intubation + iPPV on umbilical cord	1/7

Continuous numerical data are presented as mean ± SEM. iPPV, intermittent positive pressure ventilation.

### 3.2. Oxygen requirements and arterial blood gas values in the immediate transition phase

**Figure 3:**
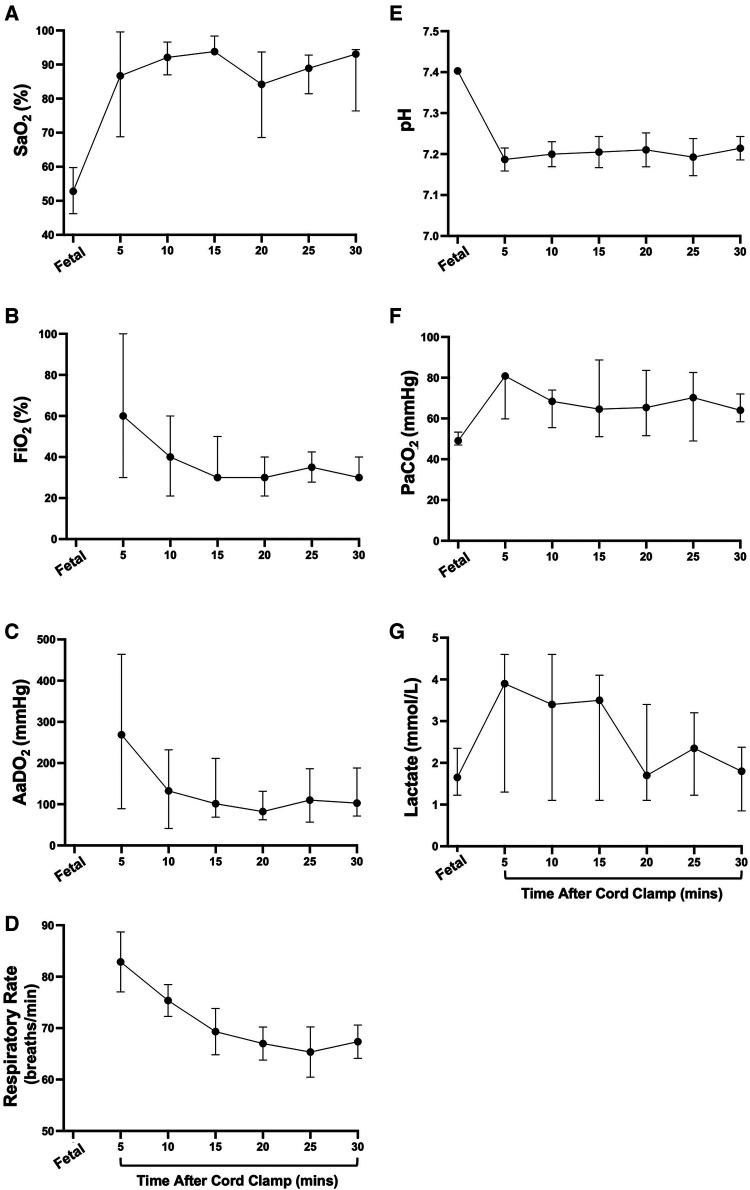
Supplemental oxygen administration, arterial oxygenation, and blood gas status immediately after birth in spontaneously breathing lambs. Arterial oxygen saturation [SaO_2_; (**A**)], fraction of inspired oxygen [FiO_2_; (**B**)], alveolar-arterial difference in oxygen [AaDO_2_; (**C**)], respiratory rate (**D**), arterial pH (**E**), partial pressure of carbon dioxide [PaCO_2_, (**F**)] and lactate (**G**) in spontaneously breathing near-term newborn lambs immediately after birth. (**A**–**C**), (**F**,**G**) are presented as median (IQR), (**D**,**E**) are presented as mean ± SEM.

At 5 min after cord clamping, median oxygen saturations were 86.7 (35.6–100)% (Figure 3A), but to achieve this a median fraction of inspired oxygen (FiO_2_) of 60 (21–100)% was required (Figure 3B), resulting in a median AaDO_2_ of 269 (37.9–610) mmHg (Figure 3C). The wide range of SaO_2_ values at 5 min reflect differences in FiO_2_ titration, where the lowest SaO_2_ values correspond to FiO_2_ being reduced prior to blood gas analysis at 5 min and higher SaO_2_ values correspond to lambs that had FiO_2_ titrated after blood gas analysis. As gas exchange (measured by AaDO_2_) and oxygenation improved over time (Figures 3A,C, respectively), supplemental oxygen (FiO_2_; Figure 3B) was reduced in turn. At 20 min, two lambs experienced desaturation episodes during oral feeding, which led to the large SEM at this timepoint. This was not an uncommon occurrence during feeding episodes, particularly in the first hour after birth, but these desaturation events were short-lived. Average respiratory rates (taken when the lambs were still) were highest at 5 min (82.9 ± 5.8 breaths/min) and gradually reduced over time (67.4 ± 3.3 breaths/min at 30 min; Figure 3D). The mean arterial pH at 5 min was 7.19 ± 0.03 and remained stable for the first 30 min after cord clamping (Figure 3E). Median partial pressure of carbon dioxide (PaCO_2_) and lactate levels peaked at 5 min (80.8 [51.1–86.0] mmHg and 3.9 [0.9–5.2] mmol/l, respectively) and remained relatively stable, although quite variable between individuals, throughout the immediate transition phase (Figures 3F,G).

### 3.3. Oxygen requirements and blood gas status in the hours following transition

**Figure 4:**
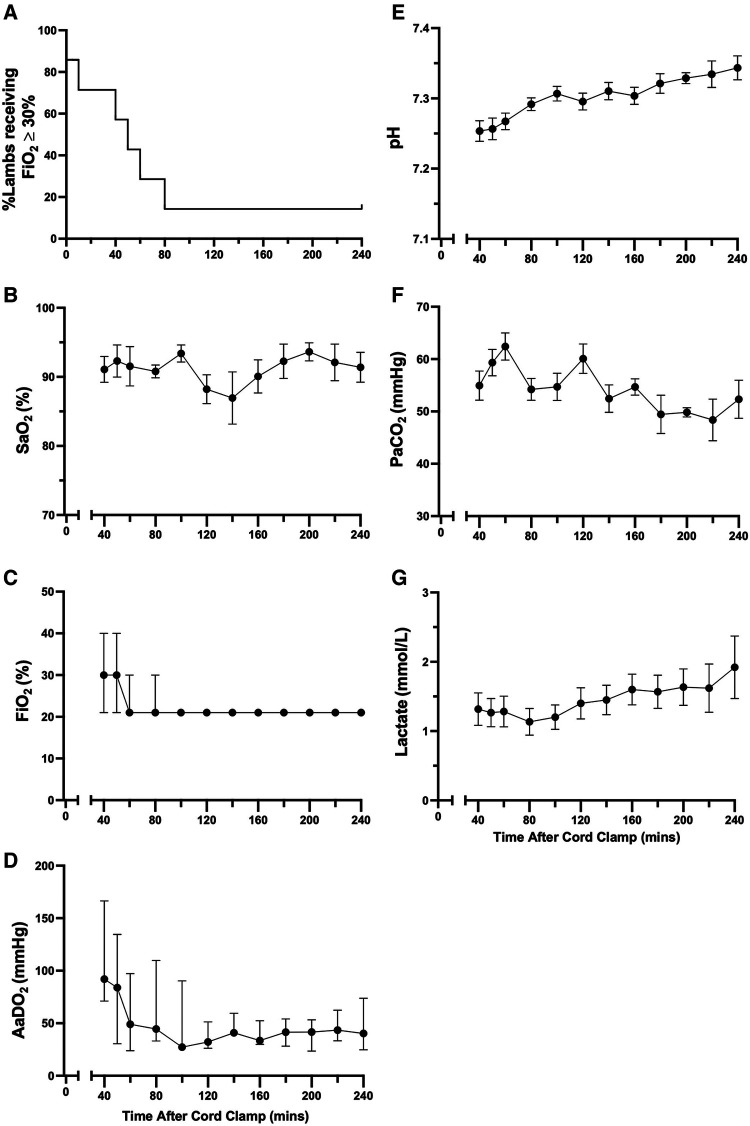
Supplemental oxygen administration, arterial oxygenation, and blood gas status in the hours after birth in spontaneously breathing lambs. Proportion of animals receiving supplemental oxygen (**A**), arterial oxygen saturation [SaO_2_; (**B**)], fraction of inspired oxygen [FiO_2_; (**C**)], alveolar-arterial difference in oxygen [AaDO_2_; (**D**)], arterial pH (**E**), partial pressure of carbon dioxide [PaCO_2_, (**F**)] and lactate (**G**) in spontaneously breathing near-term newborn lambs in the first four hours after birth. (**A**) is presented as a percentage and (**B**,**E**–**G**) are presented as mean ± SEM. (**C**,**D**) are presented as median (IQR).

The proportion of animals receiving supplemental oxygen (FiO_2 _≥ 30%; administered via nasal cannula) decreased over time until >80% of animals ceased to require support from 80 min after cord clamping (Figure 4A). One animal required supplemental oxygen to maintain SaO_2_ > 90% for the entire four hours after cord clamping (FiO_2_ 30% from 80 min onwards; Figure 4C). Both mean oxygen saturation and AaDO_2_ levels remained consistent in lambs over time (Figures 4B, D, respectively). Transient decreases in SaO_2_ were common during periods of oral feeding or excessive physical activity, as seen in Figure 4B at 140 minutes. Although mean arterial pH levels were initially low immediately following cord clamping, they gradually returned to a normal range over time (Figure 4E). Partial pressure of carbon dioxide and lactate levels were approximately stable over the first four hours after cord clamping (Figures 4F, G, respectively).

### 3.4. Respiratory patterns after birth

**Figure 5:**
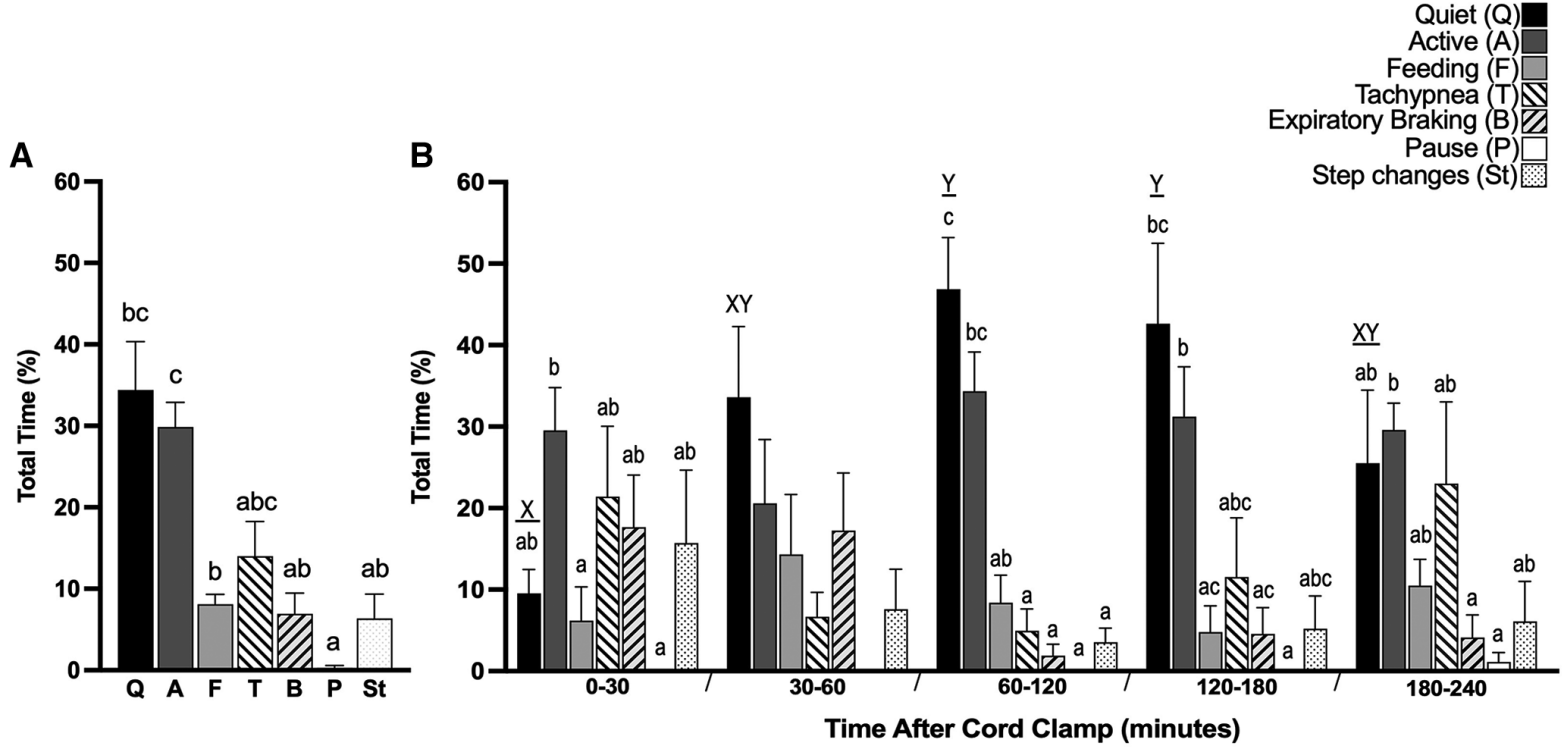
Proportion of time near-term lambs spent performing different respiratory patterns during the first 4 h after birth. Seven distinct respiratory patterns in spontaneously breathing near-term newborn lambs were identified; Quiet (**Q**), Active (**A**), Feeding (**F**), Tachypnea (**T**), Expiratory braking (**B**), Expiratory pause or hold (**P**) and Step changes in ventilation (St). Total proportion of time spent in each pattern after cord clamp [%, (**A**)] and proportion of time within each 30 min or one hour block after cord clamp [%, (**B**)] is presented. Data presented as mean ± SEM. Bars that do not share a common letter are significantly different from each other; x and y, differences within the same breathing pattern over each time block (one-way ANOVA; *p* < 0.05); a, b and c, differences between each breathing pattern within each time block (one-way ANOVA; *p* < 0.05).

**Figure 6:**
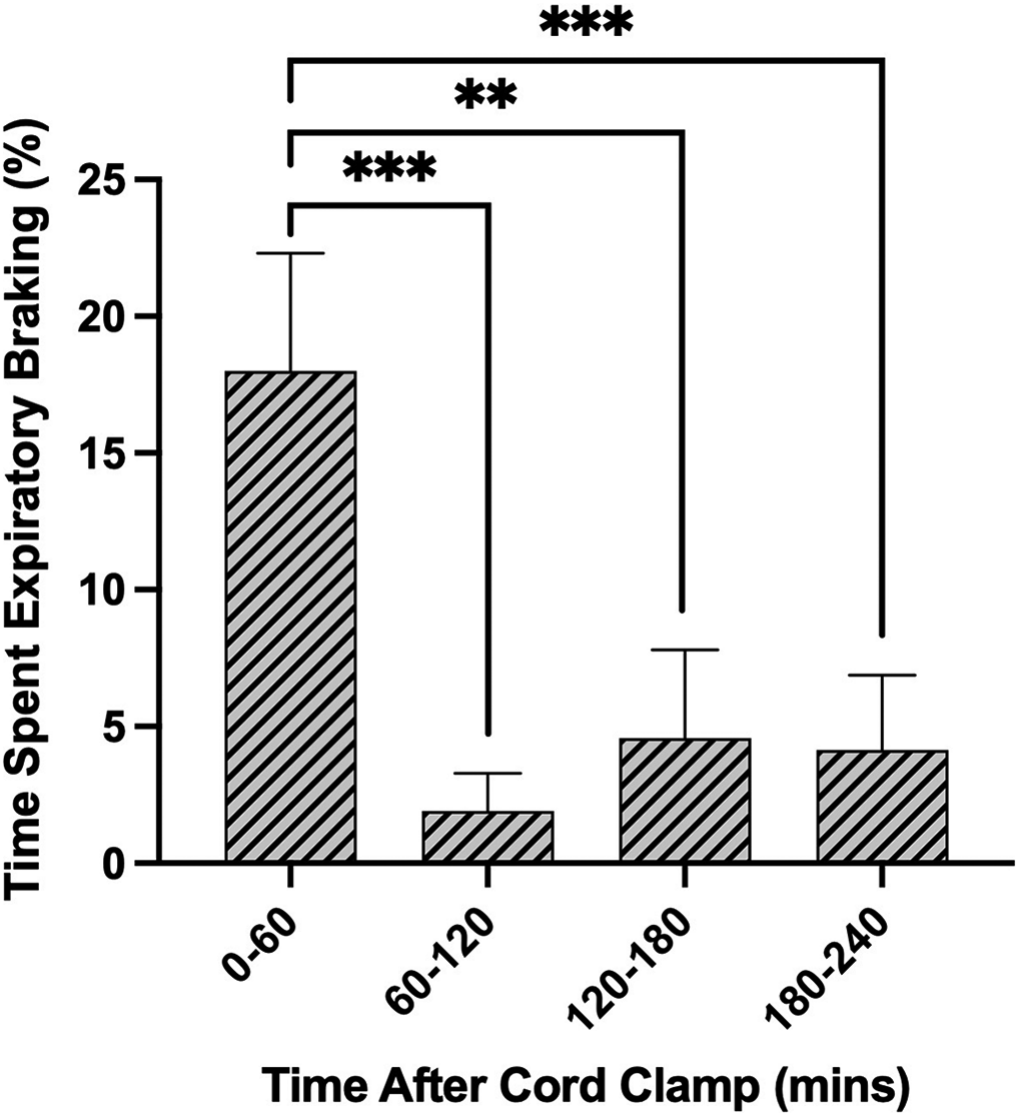
Proportion of time near-term lambs spent performing expiratory braking during the first 4 h after birth. Data are presented as mean ± SEM for every hour after cord clamping. **p < 0.01, ***p < 0.001; one-way ANOVA.

The largest proportion of time was spent in periods of quiet breathing (34.4 ± 5.9%, Figure 5A), with respiratory rates between 50 and 70 breaths/min being the most observed. Lambs also spent a large proportion of time being either active (29.9 ± 3.0%, Figure 5A) or feeding (8.1 ± 1.2%, Figure 5A), which collectively with quiet breathing, accounted for almost three quarters of the time after birth (Figure 5A). On average, lambs spent 6.9 ± 2.5% of their time making expiratory braking manoeuvres over 4 h (mostly grunting, Figure 5A), and these were significantly more common during the first hour after delivery compared with the subsequent hours (0–60 vs. 60–120 min; *p* = 0.0002, 0–60 vs. 120–180 min; *p* = 0.0011, 0–60 vs. 180–240 min; *p* = 0.0005, Figure 6). Grunting was also observed following periods of feeding (40% of feeding periods) and accounted for the majority of grunting in the latter part of the experiment. Short episodes of tachypnea were common following periods of exercise (58%; 31/53 instances of tachypnea), although some lambs experienced periods of tachypnea unrelated to physical activity. Toward the end of the experiment, lambs tended to become more active and were frequently vocalising which is consistent with the highest rate of tachypnea observed at this time.

**Figure 7:**
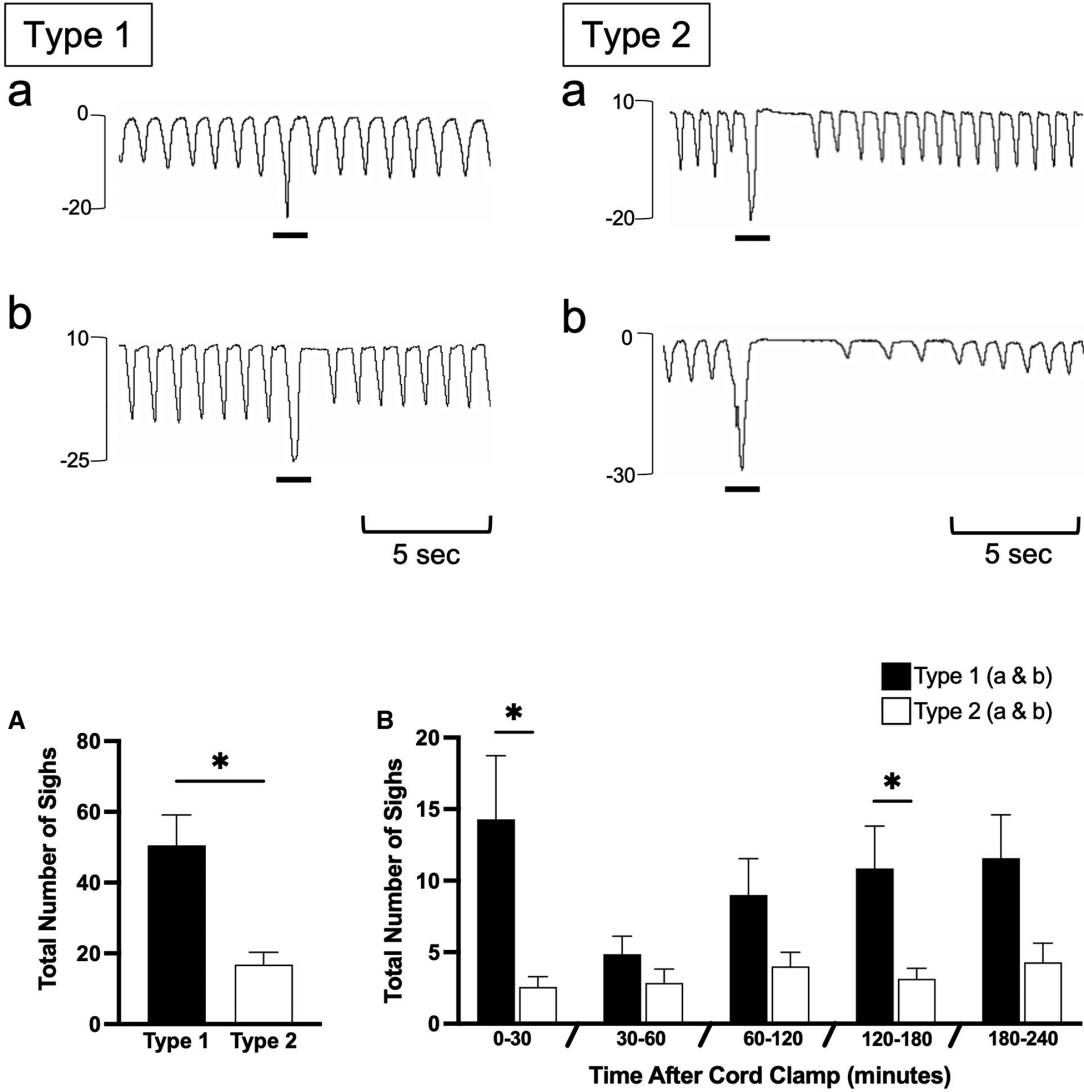
Sigh events in newborn spontaneously breathing lambs. Sighs were measured by the intrapleural pressure catheter (in cmH_2_O; inspiration is downward) and were classified as either Type 1 or Type 2 (events indicated by black underline). Type 1 sighs had little to no expiratory pause following the sigh (Type 1b and a, respectively) before immediate return of breathing. Type 2 sighs had a notable expiratory pause/apnea (>2 breath cycles in length) before immediate return of breathing (Type 2a) or a pause/apnea followed by a transient reduction in respiratory rate and breath size (Type 2b). Sighs were counted from cord clamping to 30 min, 30–60 min, and each hour thereafter (**B**) Total accumulative number of sighs is shown in (**A**) Data is presented as mean ± SEM. *p ≤ 0.05, Student's paired t-test; Type 1 vs. Type 2.

**Figure 8:**
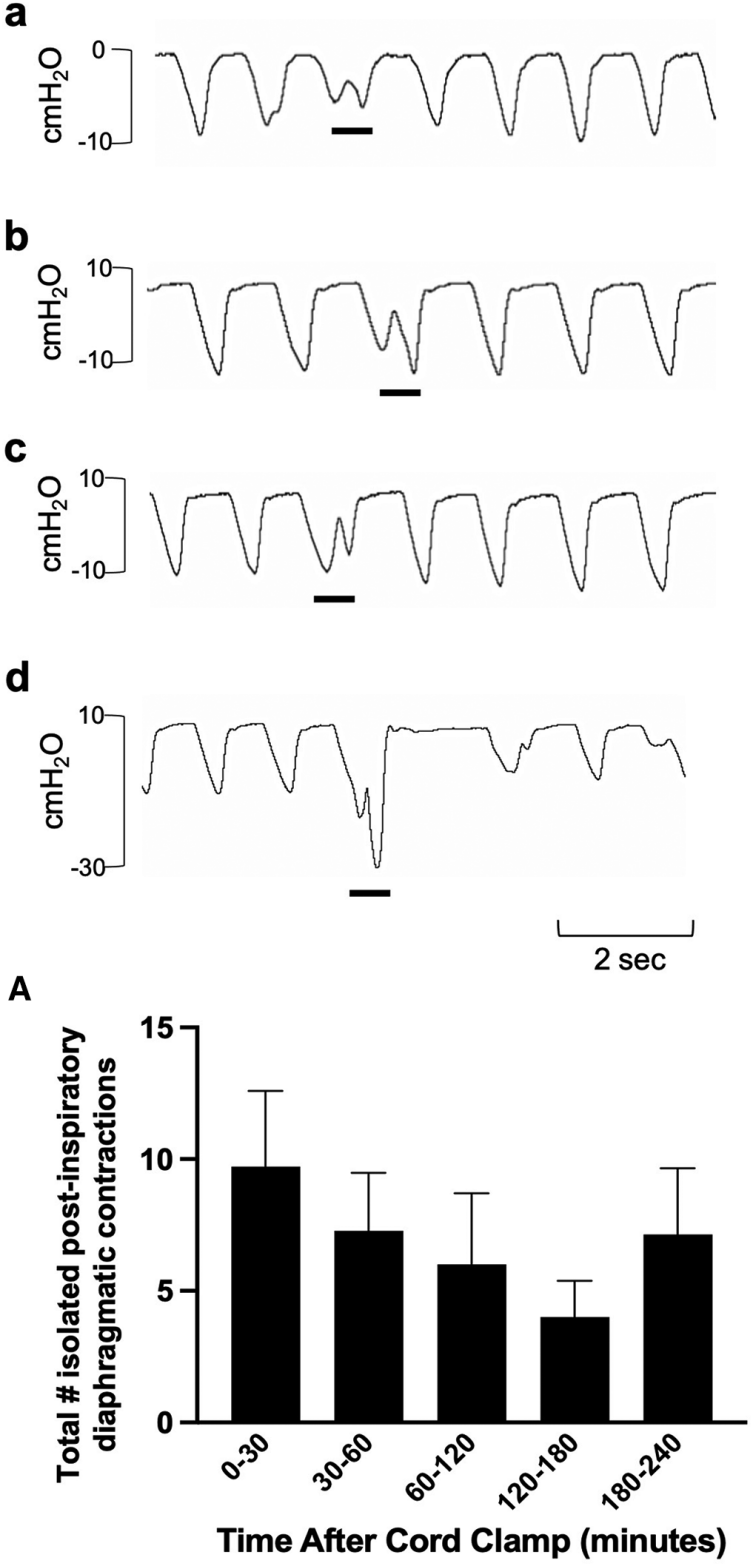
Isolated post-inspiratory diaphragmatic contractions in newborn spontaneously breathing lambs. Post-inspiratory diaphragmatic contractions were measured by the intrapleural pressure catheter (in cmH_2_O; inspiration is downward). Examples of isolated diaphragmatic contractions are shown in a-d (events indicated by black underline). Events were counted from cord clamping to 30 min, 30–60 min, and each hour thereafter (**A**) Data is presented as mean ± SEM.

Isolated breathing events, such as sighs and post-inspiratory diaphragmatic contractions, were commonly seen after delivery. Sighs followed by little to no expiratory pause (Type 1a and 1b; Figure 7) were more common compared to sighs followed by a notable expiratory pause or apnea (51 ± 9 vs. 17 ± 3 total events; Type 1 vs. Type 2; *p* = 0.014, Figure 7A). Type 2 sighs were observed in similar frequencies at each time block throughout the experiment while the frequency of Type 1 sighs appeared to be more variable over time, although changes over time were not statistically significant. No instances of complete apnea (without an associated sigh) were observed after cord clamping in this cohort of lambs. Isolated post-inspiratory diaphragmatic contractions were also noted in this cohort of lambs (Figure 8). The frequencies of these contractions appeared to change with time, although these changes were not statistically significant (Figure 8A).

## 4. Discussion

We have attempted to characterise the normal breathing patterns, and their incidence, displayed by newborn lambs during the first 4 h after birth. Lambs spent the majority (∼75%) of time either breathing quietly, being active (e.g., moving legs in an attempt to mobilise) or feeding, although for a limited amount of time they were tachypneic or made expiratory braking manoeuvres. While we consider that this range of breathing patterns reflects the normal respiratory patterns displayed by newborns lambs after birth, the incidence of each pattern is likely to vary considerably between individuals.

### 4.1. Respiratory patterns in newborns after birth

Respiratory control mechanisms, particularly in newborns, are unique and complex and difficult to study in healthy infants immediately after birth. This is because these infants rarely require respiratory support and the application of non-invasive measurement devices (e.g., facemasks) can potentially alter or even inhibit breathing (15). As such, subjective assessments of breathing behaviour are commonly used to characterise and diagnose respiratory distress (RD) in the newborn, but these assessments are inherently imprecise. In addition, the progression from normal to abnormal behaviours that indicate RD, is likely to be smooth and contiguous and simply reflect a change in the incidence or timing of behaviours that could otherwise be regarded as normal. As a result, the decision of when and how to treat term infants developing RD is difficult, largely because it is subjective and not based on definitive criteria. To improve our understanding of adverse respiratory patterns after birth and more accurately define the early stages of RD, we have described the type, incidence and timing of breathing patterns that can be regarded as normal during the first 4 h after birth.

Numerous factors input into the respiratory centre to influence respiratory breathing patterns, particularly at birth when newborns are exposed to a multitude of different stimuli. Apart from arterial oxygen and carbon dioxide levels, arousal, physical stimuli (face and body), reduced skin temperature, sound and light all impact on respiratory control, with some having negative and others positive effects on breathing (16, 17). The output from the respiratory centre, which is reflected by the breathing pattern, is an integration of all these factors, many of which have competing influences. In response to both these stimuli and the relatively high CO_2_ levels in the absence of hypoxia (Figure 3), it's not surprising that lambs were quite active during the first 30 min after birth and exhibited a relatively high level of tachypnea (∼22% of time) with only short periods of quiet breathing (<10%). We found that, on average, respiratory rates in the first 30 minutes after birth (∼60–90 breaths/min), were slightly higher than those seen in term infants delivered by CS (6, 18, 19), although our lambs were treated with caffeine. After this initial 30-minute period, the level of tachypnea decreased (to ∼5%) and the time spent in quiet breathing markedly increased (up to 50%), which coincided with stabilisation of blood gas levels. While the time spent tachypneic tended to increase and the time spent during quiet breathing tended to decrease at 3–4 h after birth, this is largely because the lambs started to become restless. The use of naloxone in newborns to treat respiratory depression, as a result of maternally administered opiates (prescribed or otherwise), has been associated with immediate onset of tachypnea following administration (20). While we anticipated that naloxone may have a similar effect in lambs, the majority of our lambs began breathing spontaneously before naloxone administration and no noticeable changes in respiratory rate were observed after receiving naloxone. In contrast, caffeine administration induced a large increase in the rate of inspiratory efforts, which was rapid in onset and similar to that observed in preterm newborn infants receiving caffeine in the delivery room (21). In our study, caffeine use was strictly limited to the first 10 min after birth and was administered as a bolus dose.

Expiratory braking manoeuvres (grunting) are a well-established feature of breathing in the immediate newborn period in both preterm and term infants (6). Grunting mostly arises due to closure of the glottis during the late expiratory phase of the breath and, at the end of the manoeuvre, expiration is completed immediately before the onset of the next inspiration. These manoeuvres are thought to aid in defending lung volume during expiration and are commonly seen in the immediate newborn period. However, as they mostly occur after the lung has aerated (22), they are unlikely to be involved in airway liquid clearance. We found that expiratory breaking manoeuvres were most common in the first hour after birth (∼20% of time), which likely coincides with the time that lung liquid has moved from the airways and into lung tissue (23). As this is known to increase lung interstitial pressures (24), it is possible that elevated hydrostatic pressures within lung tissue triggers these manoeuvres by reducing functional residual capacity (FRC) levels and activating lung volume receptors that trigger closure of the glottis (25). The increased interstitial tissue pressure may reduce FRC and activate volume receptors by reducing lung tissue compliance and increasing lung recoil. Alternatively, pulmonary oedema can induce upper airway closure in lambs days to weeks after birth, possibly via activation of juxtacapillary (J) receptors (26, 27). While vagal afferents appear to mediate both this response (26, 27) and activation of lung volume receptors (25), the exact pathways and receptors responsible for the braking we observed remain unknown. However, although juxtacapillary (J) receptors are known to be activated by pulmonary oedema, in adults these receptors trigger tachypnea (28) and so their activation may have contributed to the tachypnea we observed during the first 30 min after birth. However, if they were also involved in activating expiratory braking, as this necessarily acts to reduce breathing rates, simultaneous activation of both pathways is highly counter-productive and unlikely.

We also observed isolated diaphragmatic braking events, which have the appearance of a double inspiration, and are characterised by a second diaphragmatic contraction that occurs in the post-inspiratory phase (Figure 8). Similar to glottic closure, diaphragmatic braking is also thought to aid in preventing lung collapse (29) and often the second contraction can generate a greater pressure than the first. These occur in response to reduced lung volumes and trigger a stronger diaphragmatic contraction with lower lung volumes (29). We have also observed these events in newborn rabbits during phase contrast x-ray imaging. In rabbit kittens, the greater pressure reduction with the second contraction is caused by simultaneous contraction of the diaphragm and closure of the glottis (unpublished observations).

It is not surprising that lambs exhibited breathing behaviours that assist in maintaining FRC during the 1st hour after birth and that the incidence of braking manoeuvres reduces as the liquid is cleared from lung tissue. This has also been observed in newborn rabbits (22). While expiratory braking manoeuvres can also occur as late as 2–4 h after birth, these manoeuvres appeared to mostly occur after feeding. It is possible that this resulted from a full stomach causing rostral displacement of the diaphragm and causing a reduction in FRC, which is known to induce expiratory braking. However, it is very interesting that diaphragmatic braking occurred as single isolated events whereas grunting and expiratory braking due to a closed glottis, occurred in distinct episodes lasting many breaths. Perhaps this indicates that there is a hierarchy of lung volume defence mechanisms, with glottic closure being the first activated and the most prevalent. Nevertheless, these braking manoeuvres mostly occurred shortly after birth when the presence of airway liquid in lung tissue would be most prevalent, but also occurred during later periods when the lambs were fed.

Respiratory behaviours are also heavily influenced by whether the lambs are asleep or awake. Lambs in this experiment experienced multiple periods of both sleep and wakefulness, but in the absence of electroencephalography, we were unable to confirm whether they were asleep or awake. Instead, we used behavioural signs as a crude indicator and observed some examples of respiratory pattern changes that occurred while the lambs were sleeping. In suspected quiet sleep states (with minimal body movement), breathing rates and amplitudes were very consistent, however during suspected active sleep states (such as during rapid eye movement sleep), we observed some variabilities in respiratory rate and breath depth and an overall inconsistent breathing pattern. These may explain the majority of the “step changes in ventilation” we observed, as this breathing pattern commonly occurred when lambs were resting.

### 4.2. Study limitations

Unlike caesarean sections performed in humans, ethical requirements mandated that we use maternally administered sedatives during both the induction of spinal anaesthesia (propofol) and during delivery (midazolam). As both propofol and midazolam can cross the placenta and may cause respiratory depression in the fetus/newborn lamb, this added a level of complexity to the model. Indeed, spontaneous breathing in lambs is very sensitive to inhibitory factors such as hypoxia, anaesthetics and sedatives, which likely explains the requirement for intubation in one lamb during transition; this lamb was extubated within 10 min of intubation. Oxygen was routinely administered to avoid hypoxia-induced apnea and we found no evidence to indicate that oxygen use affected the onset of spontaneous breathing other than to stimulate breathing in lambs that were tending to become mildly hypoxic. We also tried to counter the inhibitory effects of hypoxia by transitioning lambs while attached to the umbilical cord, although this may have increased their exposure to transplacental derived midazolam. Limiting the time that the lamb is exposed to maternal sedatives may improve respiratory outcomes for these lambs, in addition to providing timely and effective respiratory stimulants and respiratory support.

## 5. Conclusion

To define adverse respiratory behaviour after birth, we first must characterise normal respiratory behaviour, which is likely to change with time after birth as the lung aerates and the liquid is cleared from lung tissue to complete the respiratory transition. We found that in our cohort of lambs, normal respiratory behaviour involves many features that are commonly associated with abnormal respiratory patterns, including tachypnea and expiratory braking. However, these patterns mostly occurred in the first hour after birth and if they occurred later, they were either associated with activity (tachypnea) or feeding (expiratory braking). Clearly, using these features to define RD in the newborn period requires much broader contextual knowledge of the infant's physiological state.

## Data Availability

The raw data supporting the conclusions of this article will be made available by the authors, without undue reservation.
